# Cytoplasmic Localization Isoform of Cyclin Y Enhanced the Metastatic Ability of Lung Cancer *via* Regulating Tropomyosin 4

**DOI:** 10.3389/fcell.2021.684819

**Published:** 2021-06-18

**Authors:** Xiaoting Zhao, Mei Jiang, Yu Teng, Jie Li, Zhefeng Li, Wende Hao, Hongyu Zhao, Chenghong Yin, Wentao Yue

**Affiliations:** ^1^Central Laboratory, Beijing Obstetrics and Gynecology Hospital, Capital Medical University, Beijing, China; ^2^Departments of Internal Medicine, Beijing Obstetrics and Gynecology Hospital, Capital Medical University, Beijing, China

**Keywords:** lung cancer, cytoplasmic isoform cyclin Y, metastasis, PFTK1, tropomyosin 4

## Abstract

Cyclin Y (CCNY) is a novel cyclin and highly conserved in metazoan species. Previous studies from our and other laboratory indicate that CCNY play a crucial role in tumor progression. There are two CCNY isoform which has different subcellular distributions, with cytoplasmic isoform (CCNYc) and membrane distribution isoform (CCNYm). However, the expression and function of CCNY isoforms is still unclear. We firstly found CCNYc was expressed in natural lung cancer tissue and cells through the subcellular distribution. Co-IP and immunofluorescence showed that both CCNYm and CCNYc could interact with PFTK1. Further studies illustrated that CCNYc but not CCNYm enhanced cell migration and invasion activity both *in vivo* and vitro. The function of CCNYc could be inhibited by suppression of PFTK1 expression. In addition, our data indicated that tropomyosin 4 (TPM4), a kind of actin-binding proteins, was down-regulated by suppression of CCNY. F-actin assembly could be controlled by CCNYc as well as PFTK1 and TPM4. As a result, CCNY was mainly expressed in lung cancer. CCNYc could promote cell motility and invasion. It indicated that CCNYc/PFTK1 complex could promote cell metastasis by regulating the formation of F-actin via TPM4.

## Introduction

Today, lung cancer is still one of the most common cancers in both men and women (Siegel et al., 2020). Despite more and more advances in cancer therapy, lung cancer still caused the greatest number of deaths (Miller et al., 2019; Carlisle et al., 2020). Identifying cancer progression-related molecules is urgently required.

Cyclin Y (CCNY, also known as CCNX, CFP1, CBCP1, and C10 or f9) is a novel cyclin and highly conserved through evolution (Liu and Finley, 2010; Liu et al., 2010). This conservation suggests that CCNY might play an important role in metazoan species. Chen and coworkers found that CCNY was ubiquitously expressed at low levels in most tissues (especially brain and lung) and at a high level in the testis (Jiang et al., 2009). Particularly, CCNY was highly expressed in human cancers, such as hepatocellular carcinoma (HCC) cells, lung cancer tissues and cell lines, glioma cells, and colorectal carcinoma cell lines (Ying-Tao et al., 2005; Xu et al., 2010; Yue et al., 2011; Shi et al., 2018). Cell growth ability was inhibited by suppressing CCNY expression in glioma cells and HCC (Xu et al., 2010; Shi et al., 2018). The results suggest that CCNY might be a new tumor biomarker for diagnosis and therapy.

As a cyclin, CCNY requires a CDK partner to regulate cellular processes. One potential CDK partner for CCNY is PFTAIRE protein kinase 1 (PFTK1, also called CDK14) (Jiang et al., 2009; Mikolcevic et al., 2012; Shehata et al., 2012; Li et al., 2014). Chen and coworkers confirmed the CCNY/PFTK1 interaction by yeast two-hybrid screening and the intracellular localization of the CCNYm-PFTK1 complex (Jiang et al., 2009). In several types of tumors, PFTK1 was involved in modulating tumor cell migration, growth and invasion (Zhu et al., 2016; Mao et al., 2017; Li et al., 2019; Sun et al., 2019; Jiang et al., 2020). The CCNY/PFTK1 complex may also play important roles in lung cancer. However, more study is required to identify the interaction between CCNYc and PFTK1.

**FIGURE 1:**
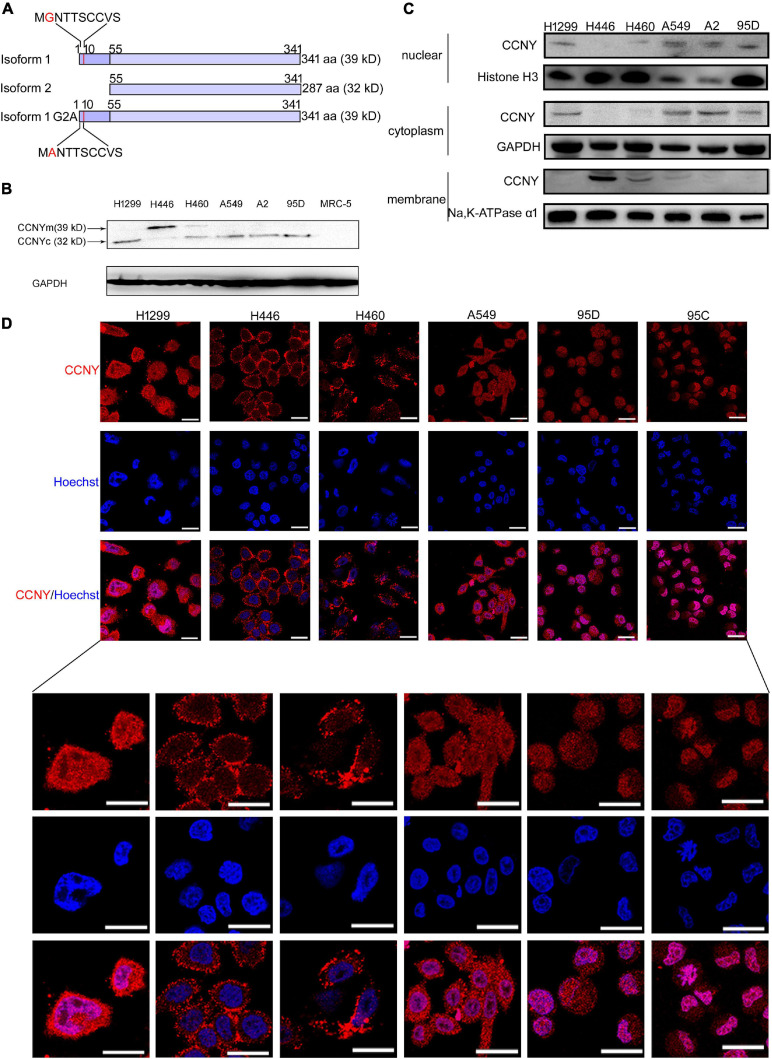
CCNYm and CCNYc were highly expressed in lung cancer cells. **(A)** A simple view of the amino acid sequence for CCNY isoforms. The letter G with red color in the CCNY isoform one sequence is the myristoylation signal motif. **(B)** The levels of CCNY and loading control GDPAH in lung cancer cells and MRC cells were determined by WB. **(C)** Western blot was done using cell cytoplasmic extracts, membrane extracts and nuclear extracts, respectively. The CCNY level in cell membrane, cytoplasm and cell nucleus was shown in the figure. **(D)** Intracellular localization of CCNY in lung cancer. Bars represent 20 μm. The red fluorescence represents CCNY protein, and the blue fluorescence is the nuclear DNA staining with Hoechst 33342.

Two isoforms of CCNY (isoform 1 and isoform 2) have been reported (Figure 1A; Li et al., 2009; Liu et al., 2010). Isoform 1 (NP_859049.2) codes a full-length 341 protein, which is membrane-localized by a myristoylation signal motif (glycine at position 2) in the N-terminal domain (Jiang et al., 2009). Isoform 2 codes an N-terminal truncated protein that lost its membrane localization. We named CCNY isoform 1 as CCNYm (CCNY membrane), while CCNY isoform 2 was named CCNYc (CCNY cytoplasm) in our article. In previous research, the function and clinical significance of CCNYm and CCNYc were not studied separately. The function of CCNY was mainly dependent on siRNAs targeted on both CCNYm mRNA and CCNYc mRNA. However, it was difficult to distinguish the function of CCNYm from that of CCNYc. Our previous research showed that the clinicopathological parameters of the NSCLC patients were not associated with the expression level of CCNY mRNA (Yue et al., 2011). We considered that CCNYm and CCNYc might have different functions in tumor cells. The functions of CCNYm and CCNYc should be determined separately.

Previously, studies on CCNY were limited to its exogenous expression. The expression level and function of CCNYc in human cells remained unknown. In this study, the subcellular location of CCNY in lung cancer tissues and cells was detected by immunofluorescence assay (IF), Western blot (WB), and immunohistochemistry (IHC). The functions of CCNYm and CCNYc were also determined by the overexpression of CCNYm and CCNYc.

In our research, we pointed out that CCNY was mainly localized in the cell cytoplasm in most of the lung cancer cells and NSCLC tissues. In H1299 and HEK293 cells, CCNYc but not CCNYm promoted cell motility and invasiveness as well as epithelial-mesenchymal transitions (EMTs). Our results indicated that the CCNYc/PFTK1 complex promoted cell migration and invasiveness by regulating microfilament assembly via TPM4 (tropomyosin 4, a member of actin-binding proteins) and RhoA.

## Materials and Methods

### Materials

G418, Fetal bovine serum (FBS), phallotoxins, RPMI 1640, DMEM, non-essential amino acids (NEAA), subcellular protein fractionation kit for cultured cells and Lipofectamine^®^ 2000 Transfection Reagent were from Thermo Fisher (Waltham, MA, United States). Transwell^®^ polycarbonate membrane cell culture inserts were bought from Corning (Tewksbury, MA, United States). Anti-vimentin (ab8978), -CCNY (ab107012), -snail (ab167615), -PFTK1 (ab224098), -β-catenin (ab6302), -TPM4 (ab181156), and -ZEB1 (ab124512) antibodies were purchased from Abcam (Cambridge, United Kingdom). The Rac1/Cdc42 activation assay kit and RhoA activation assay kit were obtained from Millipore (Billerica, MA, United States). Hoechst 33342 were obtained from Sigma (St. Louis, MO, United States). Matrigel Matrix was purchased from BD Science (San Diego, CA, United States).

### Plasmid Construction

A full-length cDNA fragment encoding CCNYm and CCNYc was amplified by RT-PCR using the primers: CCNYm-sense 5′-GA GTCGGGCTAGCTAGCTAGATGGGGAAC-3′ and antisense 5′-CATCATCTCTAAACTACGGGATCCCGCC-3′, and CCN Yc-sense 5′-GAGCACCTAGCTAGCTAGAACATGGAATTC-3′ and antisense 5′-CATCATCTCTAAACTACGGGATCCCGCC-3′. A CCNYm point mutation (G2A) was also amplified by PCR: CCNYm G2A-sense 5′-GGGCTAGCTAGCTAGATGG CTAACACTACC-3′ and antisense 5′-CATCATCTCTAAAC TACGGGATCCCGCC-3′ (Figure 1A). The PCR products were cloned into a pMD^®^19-T vector and identified by sequencing. The gene was inserted into the mammalian expression vector pEGFP-N1 vector and verified by DNA sequencing.

### Infection With Lentivirus

For cellular infection, cells were subcultured and infected by lentivirus. The shRNA sequence target for human CCNY was: 5′-AAATGTGTCGCTCTTGCAATA-3′, used in our published studies (Yue et al., 2011). Three RNA interference (RNAi) sequences targeting to PFTK1 mRNA sequence (NM_001287135.2) were used. The RNAi sequences are listed as follows: 1, 5′-GTTCATTCTTTACCACATT-3′; 2, 5′-AGGT TGCATCTTTGTTGAA-3′; 3, 5′-CGCCAACAAGTCCCAA ATT-3′. Non-silencing (NS)-small RNAi was cloned into the hU6-MCS-CMV-puromycin vector as a negative control. The virus was packaged by GeneChem (Shanghai, China).

### Transfection

Cells were transfected with Lipofectamine 2000 according to the specification. The transfected cells were screened out by using 0.25 mg/mL G418 for 4 weeks. The stable clones expressing target proteins were generated.

### Scratch Wound Assay

Cells were plated in a 6-well plate and cultured overnight before serum starvation for 24 h. After scratching with a pipette tip (20 μL), the wounds were observed and recorded at 0 and 24 h along the scratch. Cell migration ability was expressed as the migration speed of cells compared to the control cells over 24 h.

### Cell Migration and Invasion Assay

The migration and invasion assays were performed in a 24-well transwell unit containing an 8-μm pore size polycarbonate membrane. For the migration assay, after starvation for 12 h, 5 × 104 cells with 200 μL serum-free medium were added to the upper compartment of the chamber, while the lower compartment was filled with 600 μL of RPMI 1,640 supplemented with 10% FBS. After incubation at 37°C for 24 h, the tumor cells remaining inside the upper chamber were removed with cotton swabs. The cells on the lower surface of the membrane were stained with 2% crystal violet. The number of cells on the lower surface of the membrane was counted in five different microscopic fields at 200× magnification.

The invasion assay was conducted via the same procedure. Briefly, cells were seeded in the upper compartment that the membrane was coated with Matrigel Matrix (5 μg/mL). After incubation for 48 h, the number of cells on the lower surface of the membrane was counted in five microscopic fields at 200× magnification.

### Immunofluorescence Microscopy

IF assay was carried out as described in previous studies (Rausch et al., 2020; Suraweera et al., 2020). Imaging was performed with a Zeiss LSM 720 (Zeiss, Oberkochen, Germany) confocal inverted fluorescence microscope through a 40×/1.40 oil objective.

For F-actin labeling, after cell fixation and permeabilization, cells were stained with rhodamine-labeled phalloidin. Finally, nuclei were stained with Hoechst 33342. Imaging was performed with an Olympus IX81 (Olympus, Tokyo, Japan) confocal inverted fluorescence microscope through a 40× oil objective.

### High-Content Cell Analysis

A Cellomics ArrayScan HCS Reader (Thermo Fisher, Waltham, MA, United States) was used to quantify cell parameters by immunofluorescence staining. Briefly, cells were cultured in 96-well plates at 37°C for 24 h. After washing with PBS, cells were fixed in 4% paraformaldehyde and permeabilized by 0.2% Triton X-100. After blocking in 3% BSA, cells were incubated with primary antibodies overnight at 4°C and treated with TRITC-conjugated secondary antibody for 30 min at room temperature. Nuclei were stained with Hoechst 33342 for 10 min. The expression level of the corresponding protein was measured with the Cellomics ArrayScan HCS Reader using the ArrayScanTM software. The data are presented as mean ± SEM.

### GTPase Activity Assays

The activity of Rac1/Cdc42, RhoA was detected by pull-down assays. Briefly, cells were lysed and incubated with Rac1/Cdc42 assay reagent or Rho assay reagent. After washing three times with Mg^2+^ lysis buffer, the beads were collected and resuspended. WB assay were performed to detect the activity of Rac1/Cdc42, RhoA.

### Tissue Samples

Tissue microarrays (TMAs) of lung cancer and adjacent normal tissues were provided by Shanghai Outdo Biotech (Shanghai, China). The array contained 75 lung tumor specimens and 75 adjacent normal lung tissues. The 75 lung tumor specimens comprised 30 squamous cell carcinomas, 30 adenocarcinomas, seven adenosquamous carcinomas, three large-cell carcinomas, and five bronchioloalveolar carcinomas. The clinical characteristics of the patients are listed in Table 1.

**TABLE 1:** Clinical samples of tissue multi arrays.

**Characteristics**	**Number of patients**	**Positive rate of CCNY_*c*_**
**Patient age, years**		
0–60	36	0.30 (11/36)
>60	39	0.44 (17/39)
**Gender**		
Male	47	0.42 (20/47)
Female	28	0.28 (8/28)
**Histologic type**		
SCC^1^	30	0.44 (13/30)
Adenocarcinoma	30	0.37 (11/30)
Large-cell carcinoma	3	0.33 (1/3)
Bronchioloalveolar carcinoma	5	0 (0/5)
Adenocarcinoma-SCC	7	0.43 (3/7)
**Histological grade^2^**		
I	8	0 (0/8)
II	38	0.39 (15/38)
III	19	0.47 (9/19)
**Lymph node status^2^**		
Negative	6	0.33 (2/6)
Positive	58	0.40 (23/58)
**Distant Metastasis**		
Negative	75	0.37 (28/75)
Positive	0	–
**TNM Stage^2^**		
Stage I–II	6	0.33 (2/6)
Stage II–III	58	0.40 (23/58)

*^1^Squamous cell carcinoma. ^2^The characteristic of a few clinical samples was unclear.*

Lung cancer samples used in IHC assay were from the surgical specimens. The clinical characteristics of the samples are listed in Table 2. The use of lung cancer samples in this study was reviewed and approved by the Research Ethics Committee at Beijing Chest Hospital, Capital Medical University (Beijing, China). This study was performed in accordance with the Declaration of Helsinki.

**TABLE 2:** Patients and clinical characteristics.

**Characteristics**	**Number of patients**	**Positive rate of CCNY_*c*_**
**Patient age, years**		
0–60	26	0.23 (6/26)
>60	26	0.42 (11/26)
**Gender**		
Male	39	0.31 (12/39)
Female	13	0.38 (5/13)
**Smoke status**		
Non-smoker	21	0.38 (8/21)
Smoker	31	0.32 (10/31)
**Histologic type**		
SCC*	33	0.30 (10/33)
Adenocarcinoma	19	0.42 (8/19)
**Histological grade**		
I	4	0 (0/4)
II	29	0.38 (11/29)
III	19	0.32 (6/19)
**Tumor size**		
0–3 cm	12	0.42 (5/12)
>3 cm	40	0.32 (12/42)
**Lymph node status**		
Negative	27	0.33 (9/27)
Positive	25	0.32 (8/25)
**Distant metastasis**		
Negative	44	0.32 (14/44)
Positive	8	0.38 (3/8)
**TNM stage**		
Stage I	18	0.33 (6/18)
Stage II	9	0.3 (3/9)
Stage III	20	0.33 (6/20)
Stage IV	5	0.40 (2/5)

*Squamous cell carcinoma.

### *In vivo* Studies of Tumor Metastasis

All experiments were conducted with female NOD/SCID mice at 5–7 weeks old. For experimental metastasis assays, mice were injected with H1299 cells intravenously and subcutaneously. Mice were monitored for signs of metastasis, such as weight loss, subcutaneous tumors, or ascites. When these phenomena appeared, the mice were sacrificed, and tumor tissues were collected. Organ metastases were observed by gross anatomy.

All experiments involving mice were approved by the Capital Medical University Animal Care and Use Committee.

### Expression Profile for H1299-CCNY-Downregulated Cells and the Control Cells

For the expression profile analysis, H1299 CCNY KD and H1299 NC cells were collected and washed by PBS (4°C) twice. The cell pellets were lysed by TROZOL and stored at −80°C. The total mRNA of cells was extracted and the expression level of mRNA was analyzed by CapitalBio Corporation (Beijing, China).

### Statistical Analysis

All the data was expressed as the mean ± SEM. In order to comparing the means of two groups, statistic differences were analyzed with unpaired Student’s *t*-tests, where *p* < 0.05 (^∗^) was considered different, and *p* < 0.01 (^∗∗^) was considered significantly different.

## Results

### CCNY Was Mainly Detected in Cytoplasm With a High Level in Lung Cancer Cell Lines and Tissues

The level of CCNY isoforms and subcellular localization were determined by WB and IF assay. Our immunoblotting results indicated that CCNY level was much higher in lung cancer cells than that in MRC5 cells (Figure 1B). Two bands for different molecular weights (MWs) (32 and 39 kD) were detected, indicating that different isoforms of CCNY were selectively expressed in different lung cancer cells (Figure 1B). It demonstrated that 32 kD CCNY isoform was expressed in H1299, A549, A2, and 95D cells. And 39 kD CCNY isoform was detected in H446 cells. Both 32 kD CCNY isoform and 39 kD CCNY isoform were expressed in H460 cells. In order to identify the localization of CCNY isoforms, cytoplasmic extracts, membrane extracts and nuclear extracts of lung cancers were collected, respectively. As shown in Figure 1C, CCNY was mainly expressed in the cell membrane of H446 and H460 cells. And CCNY was localized in the cytoplasm in H1299, A549, A2, and 95D cells. In H460 cells, CCNY was distributed in both the cell nucleus and cell membrane. The distributions of CCNY in lung cancer cells were also detected by immunofluorescence assay. It confirmed the results of western-blot shown in Figures 1C,D. This result suggests that the distribution of CCNY was different in different lung cancer cells. In lung cancer cells, CCNY was mainly localized in the cytoplasm. This indicated that CCNYc might play more important roles in lung cancer cells.

**FIGURE 2:**
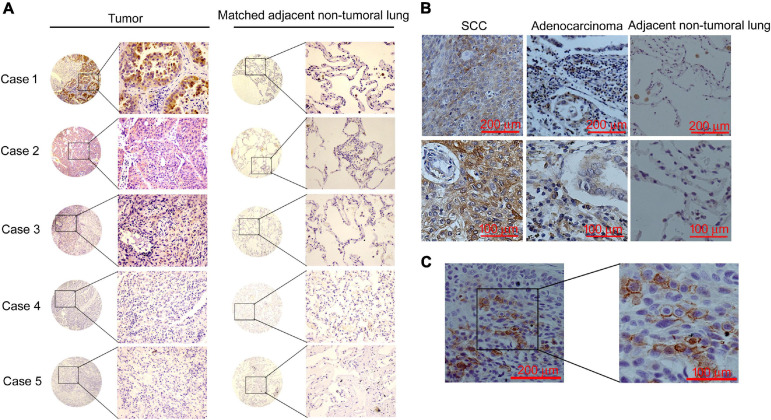
CCNY were highly expressed in NSCLC tissues. Immunohistochemical staining of CCNY. **(A)** IHC assay detecting the level of CCNY in lung cancer tissue and the paired non-tumoral lung from the TMA. CCNY was mainly localized in the cytoplasm, whereas the non-tumoral lung tissues were negative. Case 1, adenocarcinoma; case 2, squamous cell carcinoma (SCC); case 3, adenocarcinoma-SCC; case 4, large-cell carcinoma; case 5, bronchioloalveolar carcinoma (BAC). The magnification of images in circles is ×40; the magnification of images in rectangles is ×100. **(B)** Immunohistochemical examination of CCNY protein in primary NSCLC tissue and the matched non-tumoral lung tissues. Upper panel with an original magnification of ×100; lower panel with an original magnification of ×400. **(C)** IHC examination of CCNY protein in primary NSCLC tissue. CCNY was mainly distributed in the cell membrane. Original magnification, right panel, ×400; left panel, ×100.

Lung cancer TMAs were used to study the expression of CCNY in NSCLC tissues (Figure 2A and Table 1); CCNYc was highly expressed in lung cancer tissues. However, there were no positive cases in bronchoalveolar carcinoma and the adjacent normal lung tissues. We obtained similar data from paraffin-embedded lung cancer tissue sections by IHC assay (Figure 2B and Table 2). Of all the 127 cases (75 cases for TMA, 52 cases for paraffin-embedded lung cancer tissue sections), CCNYm was only found in one tumor tissue of the NSCLC cases (Figure 2C). This indicated that CCNYc might play more important roles than CCNYm in lung cancer.

### CCNYc Promotes Lung Cancer Cell Invasion and Migration in H1299

**FIGURE 3:**
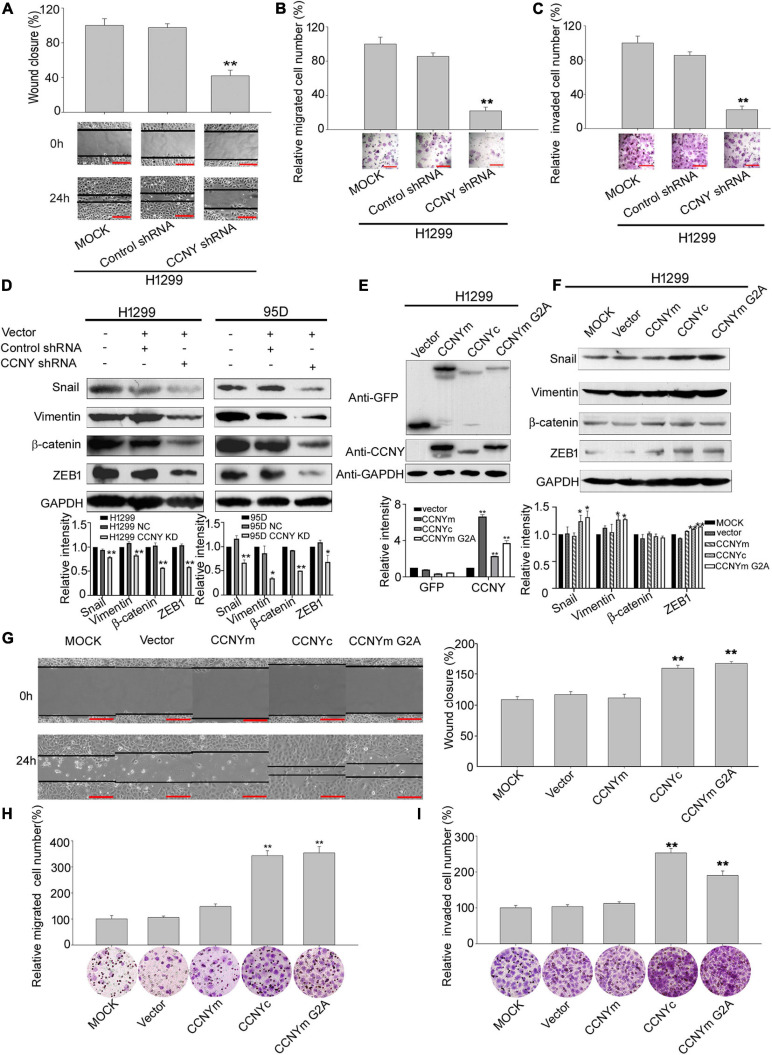
CCNYc could promote cell motility and invasiveness in H1299 cells. **(A)** Wound healing assay performed with H1299-CCNY KD and control cells over 24 h. The migration activity is shown as mean ± SEM. Bars represent 200 μm. **p < 0.01 vs. NC cells (*t*-test, *N* = 3). **(B,C)** Transwell assay performed with H1299-CCNY KD and the control cells. Cells migrated or invaded through the membrane were calculated and standardized. Bars represent 200 μm. **p < 0.01 vs. NC cells (*t*-test, *N* = 3). **(D)** The upper panel, levels of β-catenin, vimentin, ZEB1 and snail were measured by WB; the lower panel, the level of proteins was quantified by gray analysis. *p < 0.05 vs. NC cells, **p < 0.01 vs. NC cells (*t*-test). **(E)** Expression levels of GFP and CCNY were examined by immunoblotting. The levels of GFP and CCNY were quantified by gray analysis in the lower panel. **p < 0.01 vs. H1299- pEGFP-N1 cells (*t*-test). **(F)** Expression of ZEB1, vimentin, β-catenin and snail were examined by WB. The quantification of the proteins was listed in the lower panel. *p < 0.05 vs. H1299-vector cells, **p < 0.01 vs. H1299-pEGFP-N1 cells (*t*-test). **(G)** Wound healing assay performed with CCNYm, CCNYc, and CCNYm G2A upregulation cells. The migration activity is expressed as mean ± SEM. **p < 0.01 vs. H1299 cells transfected with pEGFP-N1 vector (*t*-test, *N* = 3). **(H,I)** Transwell migration assay and invasion assay were used. Bars represent 200 μm. The data are shown as mean ± SEM. **p < 0.01 vs. H1299 cells transfected with pEGFP-N1 vector (*t*-test, *N* = 3).

The role of CCNYc in lung cancer cells was explored using H1299 and 95D cells, in which CCNY was only sublocalized in the cell cytoplasm. The CCNYc was downregulated in 95D cells (95D CCNY KD) and H1299 cells (H1299-CCNY KD) by siRNA targeting CCNY (Yue et al., 2011) (Supplementary Figures 1A,B). Using scratch wound and transwell assays, we found that the migration ability was remarkably decreased by suppressing CCNYc expression (Figures 3A,B and Supplementary Figures 2A,B). The transwell invasion assay showed that CCNY knockdown strongly attenuated the cell invasive capacity (Figure 3C and Supplementary Figure 2C). These data demonstrated that CCNY downregulation depressed cell mobility and invasion in lung cancer cells.

Epithelial-mesenchymal transition is an important biologic process that cells lose their epithelial features and obtain mesenchymal characteristics (Pastushenko and Blanpain, 2019). When EMT occurred, the epithelial markers, such as E-cadherin, cytokeratin, laminin 1, were attenuated and the mesenchymal markers, such as vimentin, snail, ZEB1, twist, were acquired, with enhanced migration and invasiveness ability. The levels of EMT markers were also detected to determine the function of CCNY making during tumor metastasis (Gerashchenko et al., 2019). Western-blot data indicated that levels of vimentin, ZEB1, snail and β-catenin in CCNY KD cells were decreased compared to the control cells (Figure 3D). Immunofluorescence microscopy also illustrated that the vimentin level was decreased by stable knockdown of CCNY in H1299 and 95D cells (Supplementary Figure 2D). Similar results were obtained by high-content cell analysis (Supplementary Figure 2E). This indicated that the inhibition of CCNY expression was sufficient to induce MET (mesenchymal – epithelial transitions).

### CCNYc, Not CCNYm, Enhances H1299 Cell Migration and Invasion

H1299-CCNY KD cell strains overexpressing different CCNY isoforms with GFP tags were constructed by transfection. To confirm the function of CCNY isoforms with different subcellular localizations, CCNYm G2A, a point mutation of CCNYm, which also sublocalized in the cytoplasm, was also transfected to cells (Figures 1A, 3E and Supplementary Figures 3A,B). As indicated in Figures 3G–I, cell mobility and invasion were significantly promoted by CCNYc and CCNYm G2A but not CCNYm. The effect of CCNY isoforms on cell proliferation was also determined by MTS assay. Cell growth was only accelerated by the overexpression of CCNYm but not CCNYc in H1299 cells (data not shown). That was to say the CCNY effect on wound-healing migration was not due to the different cell growth ability. This indicated that CCNYc and CCNYm G2A promoted cell motility and invasion, while CCNYm did not affect cell migration. Moreover, CCNY distributed in the cytoplasm promoted EMT in H1299 cells (Figure 3F and Supplementary Figures 3C,D). In H1299-CCNYm cells, vimentin level was also increased comparing to H1299-pEGFPN1 cells (Supplementary Figure 3D). However, the level of other EMT markers (snail, ZEB1, β-catenin) was not altered while CCNYm was up-regulated. We thought that EMT not happened when CCNYm was overexpressed. Vimentin is one of the target genes of Wnt signaling pathway (Katoh, 2008). Since CCNYm could promote the LRP6 phosphorylation and activate Wnt signaling cascades (Davidson et al., 2009). We speculated that the vimentin level was enhanced by CCNYm via Wnt signaling pathway. Furthermore, CCNY was also overexpressed in HEK293 cells, a cell line in which we found no CCNY proteins. We obtained similar results with HEK293 cells (Supplementary Figure 4).

**FIGURE 4:**
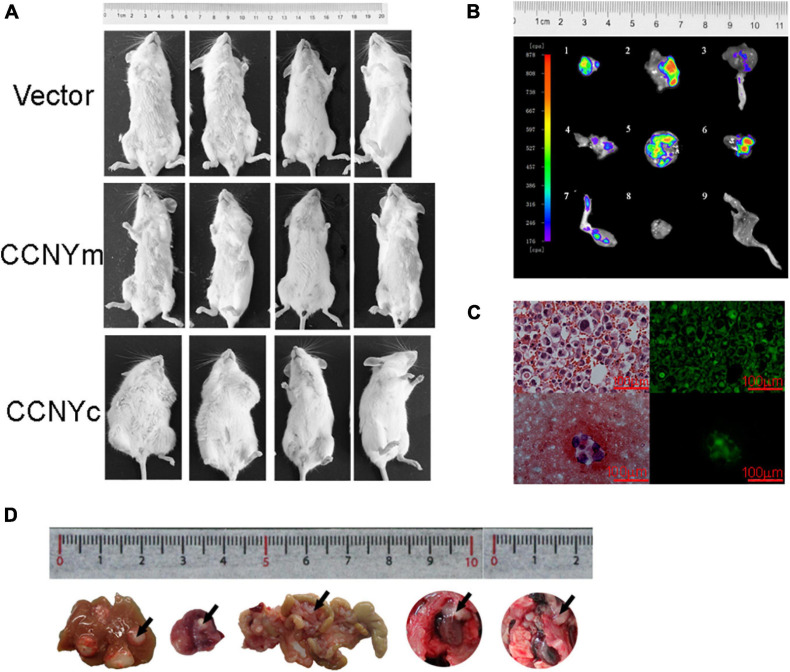
CCNYc expression promoted the metastatic potential of H1299 cells *in vivo*. **(A)** Images of mice taken 9 weeks after tail vein injection. **(B)** GFP images of metastatic tumors separated from mouse tail vein injected with H1299-CCNYc cells. 1–8, metastatic tumors (1, 2, 5, 6, abdominal lymph node; 3, 7, legs; 4, mesenteric lymph node metastasis); 8, 9, muscle tissue and leg from a mouse, used as a control. **(C)** Ascites appeared in one of eight mice injected subcutaneously with H1299-CCNYc cells. The upper panel shows a paraffin section of the ascites: tumor cells existed in the ascites, and green fluorescence was detected (right panel). The lower panel shows a smear of the ascites: tumor cells were also detected in the ascites, and green fluorescence was detected (right panel). **(D)** Images of metastatic organs (liver, celiac lymph nodes, mesentery, and pleura) separated from mice injected subcutaneously with H1299-CCNYc cells. The arrows pointed out the metastatic tumors in the organs.

To validate the biological involvement of CCNYm and CCNYc in lung cancer progression, H1299-CCNY KD cells overexpressing EGFP as well as CCNYm and CCNYc were injected into mice via the tail vein. After 9 weeks, some of the mice were found weight loss or ascites. As displayed in Figure 4A, two of four mice injected with H1299-CCNYc were obviously puffy with a lighter weight. All the twelve mice showed in Figure 4A were dissected carefully. We found that two mice with puffiness and edema developed distant metastasis, including ascites, hydrothorax, bone metastasis, abdominal lymph node metastasis, and mesenteric lymph node metastasis (Figure 4B). Conversely, mice injected with other cells showed no metastases. We also injected H1299-CCNY KD cells overexpressing CCNYm and CCNYc subcutaneously into the flank of 6-week-old NOD/SCID mice. As a control, H1299-CCNY KD cells transfected with pEGFPN1 alone were also injected subcutaneously into the mice. Three groups with eight mice each were used in this study. The mice began to develop ascites, weight loss, or inactivity after 5 weeks. Four of eight mice injected with H1299-CCNYc cells developed distant metastases. Metastatic tumor cells were observed in the liver, mesentery, lymphonodus, and pleura, as well as ascites in the mice injected with H1299-CCNYc cells (Figures 4C,D). The green fluorescence of GFP remained in the tumor cells (Figure 4C). However, no metastases were found in any other group. These combined data demonstrate that CCNYc overexpression leads to increased metastatic behavior of H1299 lung cancer cells. Above all, they indicated that the distribution of CCNYc was critical for lung cancer metastasis *in vitro* as well as *in vivo*.

### PFTK1 Was Involved in the Cellular Process Caused by CCNYc

**FIGURE 5:**
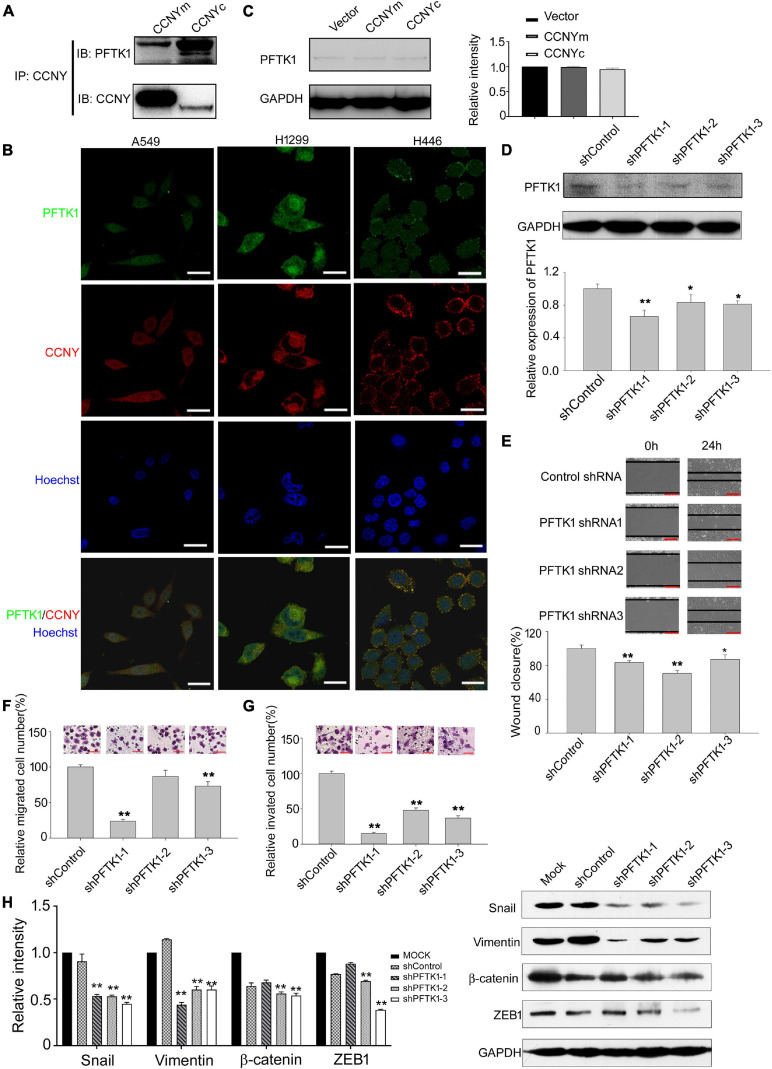
PFTK1 was involved in CCNYc signal pathway. **(A)** Co-IP assay was done using H1299 CCNYc and H1299 CCNYm cells. Co-IP demonstrated the binding ability of CCNY and PFTK1. **(B)** Co-localization of PFTK1 and CCNY in human lung cancer cells. PFTK1 and CCNY were marked by immunofluorescence staining (IF). Bars represent 10 μm. **(C)** The level of PFTK1 in CCNY up-regulated H1299 cells was examined by WB (left panel) and quantified by gray analysis (right panel). **(D)** H1299 CCNYc cells were infected by recombinant lentivirus carrying shRNA targeting to PFTK1 mRNA. PFTK1 mRNA expression was inhibited by PFTK1-shRNA1-3. The level of PFTK1 was detected by WB (upper panel) and high-content cell analysis (lower panel). *p < 0.05 vs. shControl H1299-CCNYc cells, **p < 0.01 vs. shControl H1299-CCNYc cells (*t*-test). **(E)** Wound healing assay performed with PFTK1 downregulation cells. The migration activity is expressed as mean ± SEM. **p < 0.01 vs. H1299-CCNYc cells expressing control shRNA (*t*-test, *N* = 3). **(F,G)** Transwell migration assay and invasion assay of H1299. The data are shown as mean ± SEM. **p < 0.01 vs. H1299-CCNYc cells expressing control shRNA (*t*-test, *N* = 3). Bars represent 200 μm. **(H)** Expression of ZEB1, vimentin, β-catenin and snail and was examined by WB (right panel). The left panel, the level of proteins was quantified by gray analysis. *p < 0.05 vs. H1299-CCNYc cells expressing control shRNA, **p < 0.01 vs H1299-CCNYc cells expressing control shRNA (*t*-test).

Although PFTK1 is a potential CDK partner for CCNYm (Liu et al., 2010; Sun et al., 2014), we still did not know whether CCNYc interacted with PFTK1 in lung cancer cells. Co-IP assay was done using H1299-CCNYm cells and H1299-CCNYc cells. Based on our Co-IP results, both CCNYm and CCNYc combined with PFTK1 in H1299 cells (Figure 5A and Supplementary Figure 5). IF assay was also used to detect the distribution of endogenous CCNY and PFTK1 in lung cancer cells. Cells expressing CCNYc (A549 and H1299) as well as CCNYm (H446) were all used. Consequently, the endogenous CCNY and PFTK1 are located in the same subcellular compartments in lung cancer cells (Figure 5B). This confirmed that PFTK1 was a CCNYm/CCNYc partner in lung cancer cells.

In the following experiments, the role of PFTK1 in the CCNYc signal pathway was determined. Consequently, the PFTK1 expression level was not influenced by the upregulation of CCNYm and CCNYc (Figure 5C). Downregulation of PFTK1 caused by shRNA significantly decreased the ability of H1299-CCNYc cell mobility and invasion (Figures 5D–G). As shown in Figure 5H, EMT was inhibited by the downregulation of PFTK1 in H1299-CCNYc. Above findings suggest that the CCNYc-promoted cell potency of metastasis was inhibited by the downregulation of PFTK1.

### CCNYc/PFTK1 Complex Modulates Intracellular Cytoskeleton *via* TPM4

**FIGURE 6:**
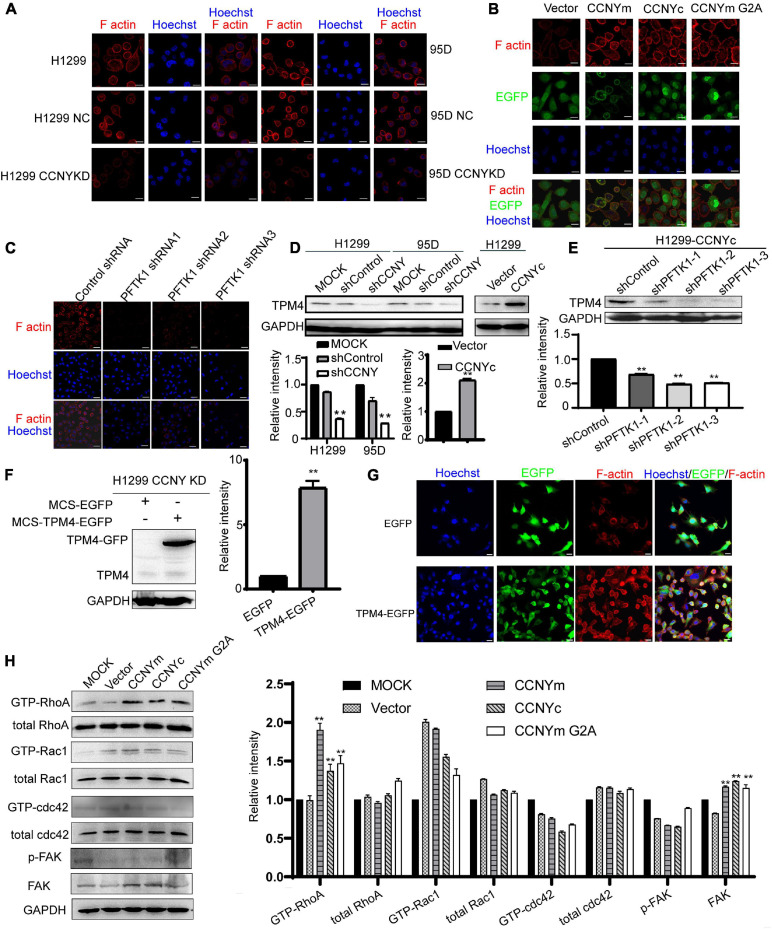
CCNYc/PFTK1 complex promoted the assembly of F-actin via TPM4. **(A–C)** F-actin was marked by IF staining. Bars represent 20 μm. **(D)** Expression level of TPM4 was shown in the figure (upper panel). The quantification of TPM4 level was done by gray analysis (lower panel). **p < 0.01. **(E)** TPM4 level in PFTK1 down-regulated H1299 CCNYc cells. Upper panel, the picture of WB, lower panel, the quantification of the TPM4 level. **p < 0.01 vs. H1299-CCNYc cells expressing control shRNA. **(F)** TPM4 level was detected by WB. The quantification of TPM4 was shown in the right panel. **p < 0.01. **(G)** F-actin was marked by phalloidin conjugating to TRITC. Bars represent 20 μm. **(H)** RhoA activity was regulated by CCNY. GTPase activity assay was used to determine the activated level of (GTP-) Rac1, RhoA, and Cdc42. The phosphorylated FAK on Y397, as well as the FAK level, were examined by immunoblotting. The protein levels were quantified by gray analysis and showed in the right panel. **p < 0.01 vs. H1299 cells transfected with pEGFP-N1 vector.

Cell molibity and invasiveness involve the regulation of the cytoskeleton (Gerashchenko et al., 2019; Klingler-Hoffmann et al., 2019). The effect of CCNYc on cell F-actin assembly was determined, and phalloidin was used to label F-actin. The F-actin level was obviously decreased by downregulation of CCNY and enhanced in CCNYm, CCNYc, and CCNYm G2A overexpression cells (Figures 6A,B and Supplementary Figures 6A–C). More filopodia were found in CCNYc and CCNYm G2A upregulated cells. The F-actin level in PFTK1 knockdown cells was noticeably inhibited by suppressing PFTK1 expression in H1299-CCNYc cells (Figure 6C and Supplementary Figure 6D).

Expression profile analysis of H1299-CCNY-downregulated cells was used to identify the cytoskeletal-related proteins regulated by CCNY (CapitalBio Corporation, Beijing, China, and Supplementary Data). The TPM4, a member of actin-binding proteins (Khaitlina, 2015), was downregulated by suppression of CCNY. Two repeats were done in the expression profile analysis. And the ratio of H1299-CCNY KD/H1299 NC were 0.4033 and 0.3006, respectively. *Tpm4* is one gene type of tropomyosin isoforms. Tropomyosin is a major regulatory protein of cytoskeleton and is localized along actin filaments modulating the interaction of actin and myosin (Khaitlina, 2015; Janco et al., 2020). As illustrated in Figure 6D, the level of TPM4 was decreased in CCNY downregulating cells and enhanced in CCNY upregulated cells. In CCNYc up-regulated H1299 cells, TPM4 expression was inhibited by the suppression of PFTK1 (Figure 6E). It indicated that TPM4 played important roles in CCNY caused cellular process. In order to explore the role TPM4 made during the CCNY cellular process, H1299 CCNY KD cells were infected by recombinant lentivirus carrying MCS-EGFP or MCS-TPM4-EGFP. TPM4 labeling with EGFP was overexpressed in H1299 CCNYKD cells (Figure 6F). The F-actin level was obviously increased due to the up-regualtion of TMP4 (Figure 6G).

The activity of Cdc42, Rac1, and RhoA in CCNY upregulated cells was also detected by pull-down assay. The GTP-RhoA level was increased, while CCNY was overexpressed in H1299 cells (Figure 6H). The level of GTP-Rac1 and GTP-Cdc42 was hard to detect in our pull-down assay. According to the western blot assay, the GTP-Rac1 level and GTP-Cdc42 level was not affected by the expression CCNY isoforms. The level of phosphorylated focal adhesion kinase (FAK Y397), a focal adhesion-associated protein kinase regulating cellular adhesion, was not affected by the CCNY level. We obtained similar results for HEK293 cells (Supplementary Figure 7).

## Discussion

**FIGURE 7:**
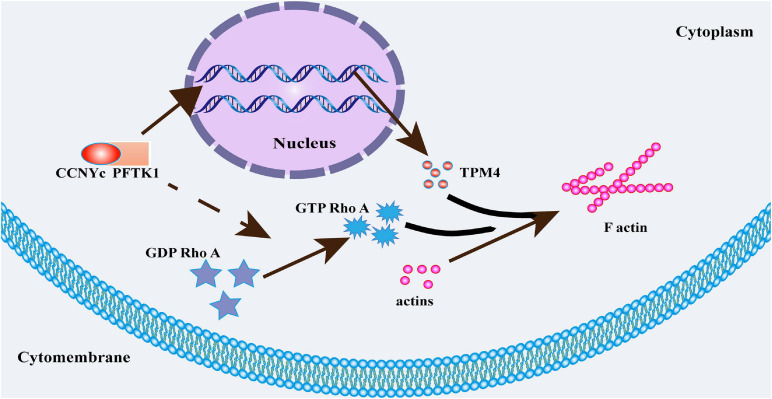
Signal pathway of CCNYc promoting cell metastasis ability.

In this study, it demonstrated that CCNY was highly expressed in lung cancer. Our results indicated that two natural isoforms existed with different subcellular distributions of CCNY expressed in human cells. Moreover, CCNY was mainly localized in the cell cytoplasm of lung cancer. We speculated that CCNYc played more important roles in NSCLC. Consequently, the CCNYc/PFTK1 complex promoted cell migration and invasion by modulating the cytoskeletal structure via TPM4, both *in vivo* and *in vitro* (Figure 7). However, CCNYm minimally affected cell migration and invasion in H1299 cells.

Cyclin Y was first discovered as a membrane-associated protein in *Drosophila* (Liu and Finley, 2010). Recent studies indicated that CCNY has another isoform distributed in the cell cytoplasm, which we called CCNYc (Li et al., 2009). However, whether CCNYc protein exists in human cells has not yet been reported. Based on the different subcellular localization and different MWs of CCNYm and CCNYc, we reported for the first time that both CCNYc and CCNYm were expressed in lung cancer with a high level. In lung cancer cells we detected, CCNY was mainly localized in the cytoplasm. In H460 cells, CCNY was distributed in both the cell cytoplasm and membrane. In H446 cells, CCNY was mainly distributed in the cell membranes. The function of CCNYm expressed in H446 and H460 cells remains unknown. This must be investigated in the future. In lung cancer tissue, CCNY was also mostly localized in the cytoplasm except for one case. It is possible that CCNYc plays more important roles in lung cancer cells. The clinical significance of CCNYm should be analyzed in the future.

The function of CCNY in cell proliferation was thoroughly studied; CCNY phosphorylates LRP6 (lipoprotein receptor-related protein six) at G2/M and induces Wnt/β-catenin signaling for regulating a mitotic program in HEK293 cells (Davidson et al., 2009). In many tumors, CCNY was involved in regulating cell growth, such as in HCC, renal cancer, breast cancer, and ovarian cancer (Liu et al., 2016; Yan et al., 2016; Shi et al., 2018; Si et al., 2019). It appears that CCNY promotes cell cycle progression via different signal pathways. Our previous study indicated that the level of serum CCNY, as well as serum anti-CCNY autoantibody, was related to distant metastasis in NSCLC (Ma et al., 2013, 2019). In HCC and ovarian cancer, CCNY also promoted cell migration and invasion (Liu et al., 2016; Shi et al., 2018). Based on our results, CCNYc, as well as CCNYm G2A, promoted lung cancer metastasis. This demonstrated that the function of CCNY depended on the subcellular localization. That is, the cell potency of cell metastasis was regulated by CCNY distributed in the cytoplasm.

A potential CDK partner for CCNY was PFTK1 (Liu et al., 2010; Sun et al., 2014). In a previous study, PFTK1 was recruited to LRP6 at the membrane by interacting with CCNYm, which is enriched at the plasma membrane, and the PFTK1/CCNY-LRP5/6 complex was formed at G2/M (Davidson et al., 2009; Wang et al., 2016). In cultured cells, the binding of CCNY to PFTK1 enhanced the PFTK1 kinase activity (Jiang et al., 2009). According to our results, both CCNYc and CCNYm interacted with PFTK1 in H1299 cells. Moreover, CCNYc and PFTK1 were co-localized in the cell cytoplasm. This indicated that PFTK1 was a CDK partner of CCNYc in lung cancer cells. The ability of cell mobility and invasiveness was significantly inhibited by downregulation of PFTK1 in CCNYc-overexpressed cells. This suggests that upregulation of CCNYc promotes the cell metastasis potency and causes EMT by binding to PFTK1.

The active formation of actin stress fibers plays important roles in cell mobility and invasion (Gerashchenko et al., 2019; Klingler-Hoffmann et al., 2019). In HCC, CCNY/PFTK1 promotes actin polymerization by activating non-canonical Wnt signaling via Rho GTPase activation (Sun et al., 2014). Oncogenic PFTK1 conferred cell motility by promoting the formation of actin stress fibers in HCC (Leung et al., 2011a,b). We supposed that CCNYc/PFTK1 promoted cell motility by affecting intracellular cytoskeletal components. The expression profile analysis data for H1299 and CCNY-downregulated cells demonstrated that TPM4, an actin-binding protein, was regulated by the expression of CCNY. This TPM4 is an important part of the cytoskeleton (Manstein and Mulvihill, 2016). It stabilizes the microfilament structure and regulates the functions of other actin-binding proteins (Khaitlina, 2015). In our previous studies, TPM4 accelerated cell migration by promoting the polymerization of actin monomers (Zhao et al., 2019). In HCC, the cell motility was also suppressed by the downregulation of TPM4 (Sheng and Chen, 2020). We considered that CCNYc/PFTK1 regulated the cell cytoskeletal structure via TPM4. We then found that filopodia, as well as F-actin levels, induced in CCNY knockdown cells, while they were enhanced by upregulation of CCNYc. Moreover, F-actin formation was inhibited by downregulation of PFTK1 in CCNYc-overexpressed H1299 cells.

The Rho family of GTPase plays important roles in regulating intracellular actin dynamics (Jung et al., 2020). Actin stress fiber assembly is regulated by the Rho GTPase family to modulate cell movement. The most common members of the Rho GTPase family were Cdc42, Rac1, and RhoA. In this study, we also detected the effect of CCNYc on the activity of Cdc42, Rac1, and RhoA. Consequently, the GTP-RhoA level was increased in either CCNYm or CCNYc-overexpressing cells. This indicated that CCNYc might modulate filamentous actin organization via activating RhoA. Combined, CCNYc/PFTK1 promoted cell migration and invasiveness by F-actin remodeling via TPM4 and RhoA. The F-actin level and RhoA activity were also increased in CCNYm upregulation cells; however, the cell motility and invasion activity were not enhanced. We considered that CCNYm was a membrane-associated protein. The overexpression of membrane protein might affect the structure of the cytoskeleton via other signal pathways that differ from those of CCNYc.

## Conclusion

In our study, we found that two isoforms of CCNY with different subcellular distributions were highly expressed in lung cancer cells and NSCLC tissues. Moreover, CCNY was mainly distributed in the cytoplasm of NSCLC tissues. Both *in vivo* and *in vitro*, CCNYc (including CCNYm G2A) but not CCNYm promoted tumor cell metastasis. In lung cancer cell lines, the CDK partner of CCNYm and CCNYc was PFTK1. The effect of CCNYc was abolished by stable downregulation of PFTK1. The F-actin assembly was regulated by CCNYc via the expression of TPM4. Our combined findings demonstrate that the CCNYc/PFTK1 complex promoted cell metastasis potency by regulating cytoskeletal assembly via TPM4 and RhoA (Figure 7).

## Data Availability Statement

The original contributions presented in the study are included in the article/Supplementary Material, further inquiries can be directed to the corresponding author/s.

## Ethics Statement

The studies involving human participants were reviewed and approved by the Research Ethics Committee at Beijing Chest Hospital, Capital Medical University. The patients/participants provided their written informed consent to participate in this study. The animal study was reviewed and approved by the Capital Medical University Animal Care and Use Committee. Written informed consent was obtained from the individual(s) for the publication of any potentially identifiable images or data included in this article.

## Author Contributions

WY, CY, XZ, and MJ designed and supervised the whole study. YT, JL, and ZL did lots of work on data collection and curation. WH and HZ mainly maintained the cell lines used in the research. All authors contributed to the writing of this manuscript.

## Conflict of Interest

The authors declare that the research was conducted in the absence of any commercial or financial relationships that could be construed as a potential conflict of interest.
