# Supplementary material for: Cytoplasmic Localization Isoform of Cyclin Y Enhanced the Metastatic Ability of Lung Cancer via Regulating Tropomyosin 4
Source: Front Cell Dev Biol. 2021 Jun 18;9:684819. doi: 10.3389/fcell.2021.684819 (PMC8250429; doi:10.3389/fcell.2021.684819)
Supplement: Supplementary file 2 [file Data_Sheet_2.docx]

Supplementary Material

# Supplementary Data

The supplementary figures and figure legends mainly including the subcellular distribution of exogenous CCNYm and CCNYc in H1299 and HEK293 cells, the roles of CCNYc played in EMT, and the function of CCNYc in HEK293 cells.

# Supplementary Figures

#
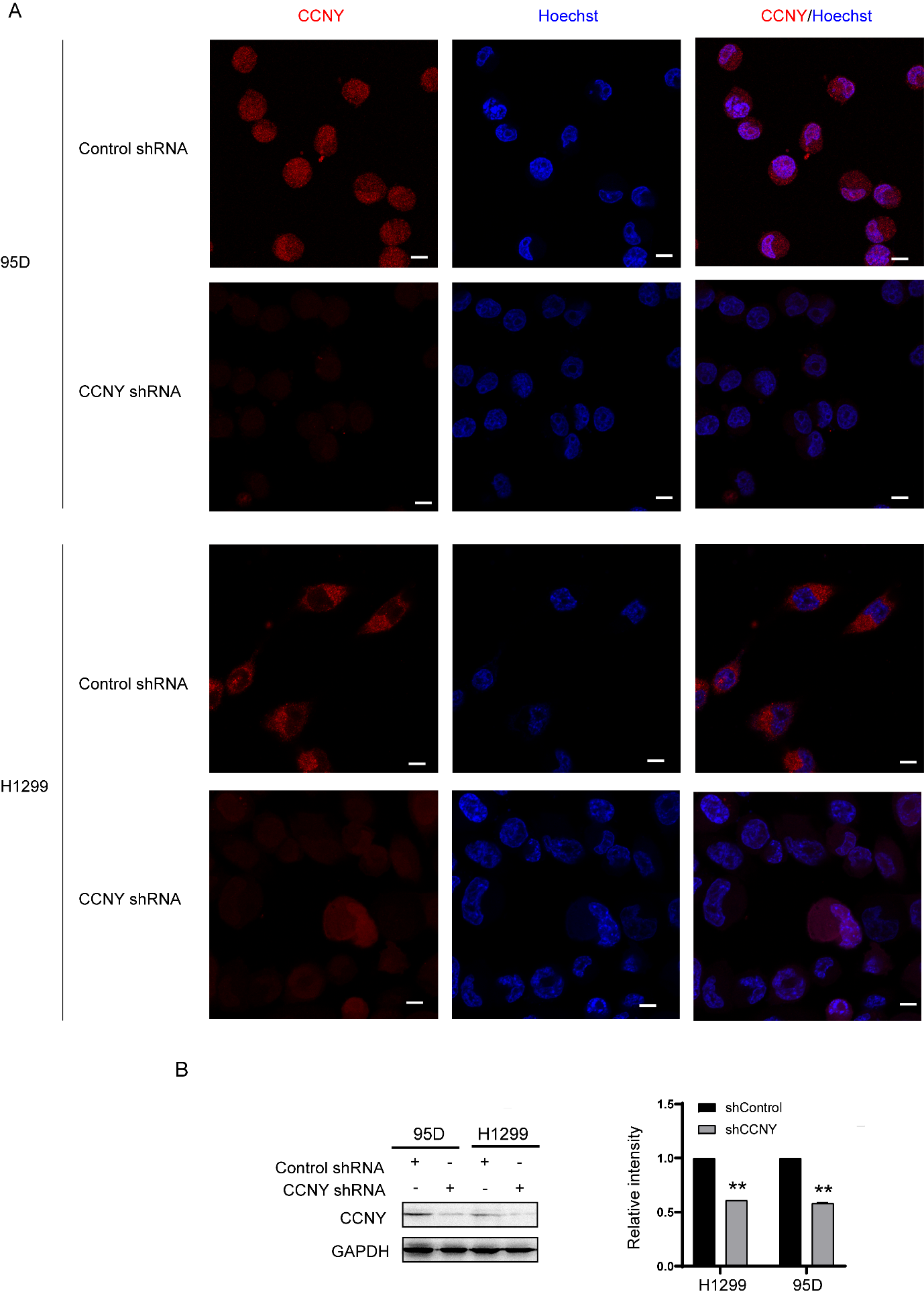


**Supplementary Figure 1.** CCNY was downregulated by CCNY shRNA in H1299 and 95D cells. A, Immunofluorescence staining of CCNY in H1299 and 95D cells. It appeared that CCNY expression was suppressed by the shRNA targeting CCNY mRNA. Bars represent 10 μm. B, CCNY level was detected by WB (the left panel) and it was quantified by grey analysis shown in the right panel. ** p <0.01 vs. NC cells.


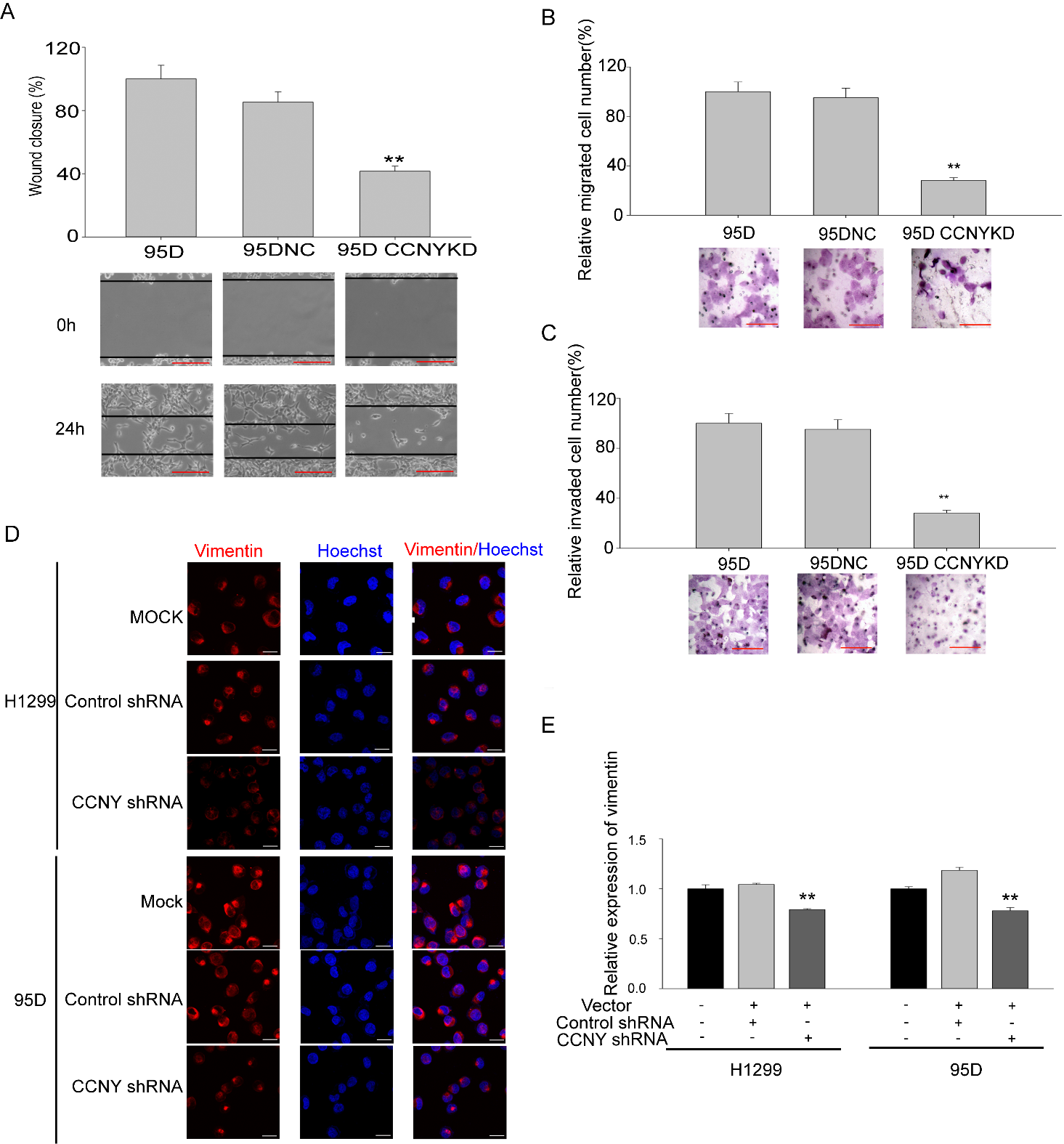


**Figure 2** CCNYm could promote cell mobility and invasiveness in lung cancer. A, Wound healing assay to determine the ability of cell migration. The migration activity is expressed as mean ± SEM. ** p <0.01 vs. NC cells (t-test, N = 3). Bars represent 200 μm. B, C, Transwell migration assay and invasion assay were performed with 95D CCNY KD cells as well as the control cells. Bars represent 200 μm. ** p <0.01 vs. NC cells (t-test, N = 3). D, the expression of vimentin was inhibited by downregulation of CCNY in lung cancer cells. Immunofluorescence staining of vimentin in H1299-CCNY KD and 95D CCNY KD cells as well as the control cells. Bars represent 20 μm. E, the vimentin level was measured by high-content cell analysis. The fluorescence intensity was quantified with a Cellomics ArrayScan HCS Reader (Thermo Fisher, Waltham, MA, USA) using the ArrayScanTM software. The data are shown as mean ± SEM. **p <0.01 (compared with cells expressing control shRNA, t-test, N = 3).


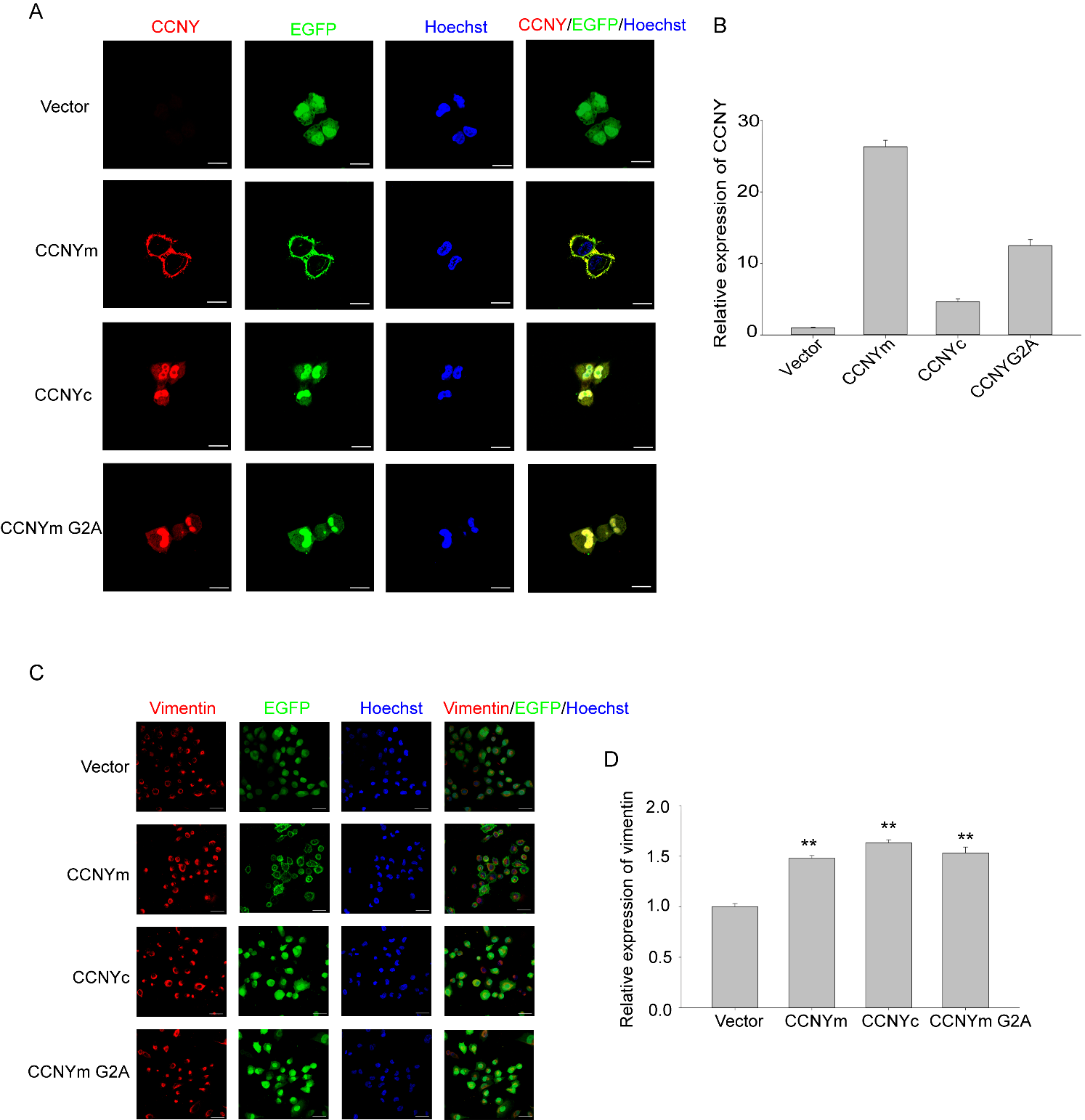


**Supplementary Figure 3** Vimentin level was upregulated by the overexpression of CCNY. A, CCNYm, CCNYc, and CCNYm G2A were successfully overexpressed in H1299 cells. H1299 cells were transfected with pEGFP-N1, pEGFP-N1-CCNYm, pEGFP-N1-CCNYc, and pEGFP-N1-CCNYm G2A. The expression of CCNY was detected by IF. Bars represent 20 μm. B, CCNY expression level was detected by high-content cell analysis. The data are shown as mean ± SEM. **p < 0.01 (compared with H1299 transfected with pEGFP-N1 vector, *t*-test, N = 3). C, Immunofluorescence staining of vimentin in H1299 cells. Bars represent 20 μm. D, Vimentin level was measured by high-content cell analysis. The vimentin level was normalized to H1299 cells transfected with the pEGFP-N1 vector. The data are shown as mean ± SEM. **p <0.01 (compared with cells transfected with pEGFP-N1 vector, t-test, N = 3).


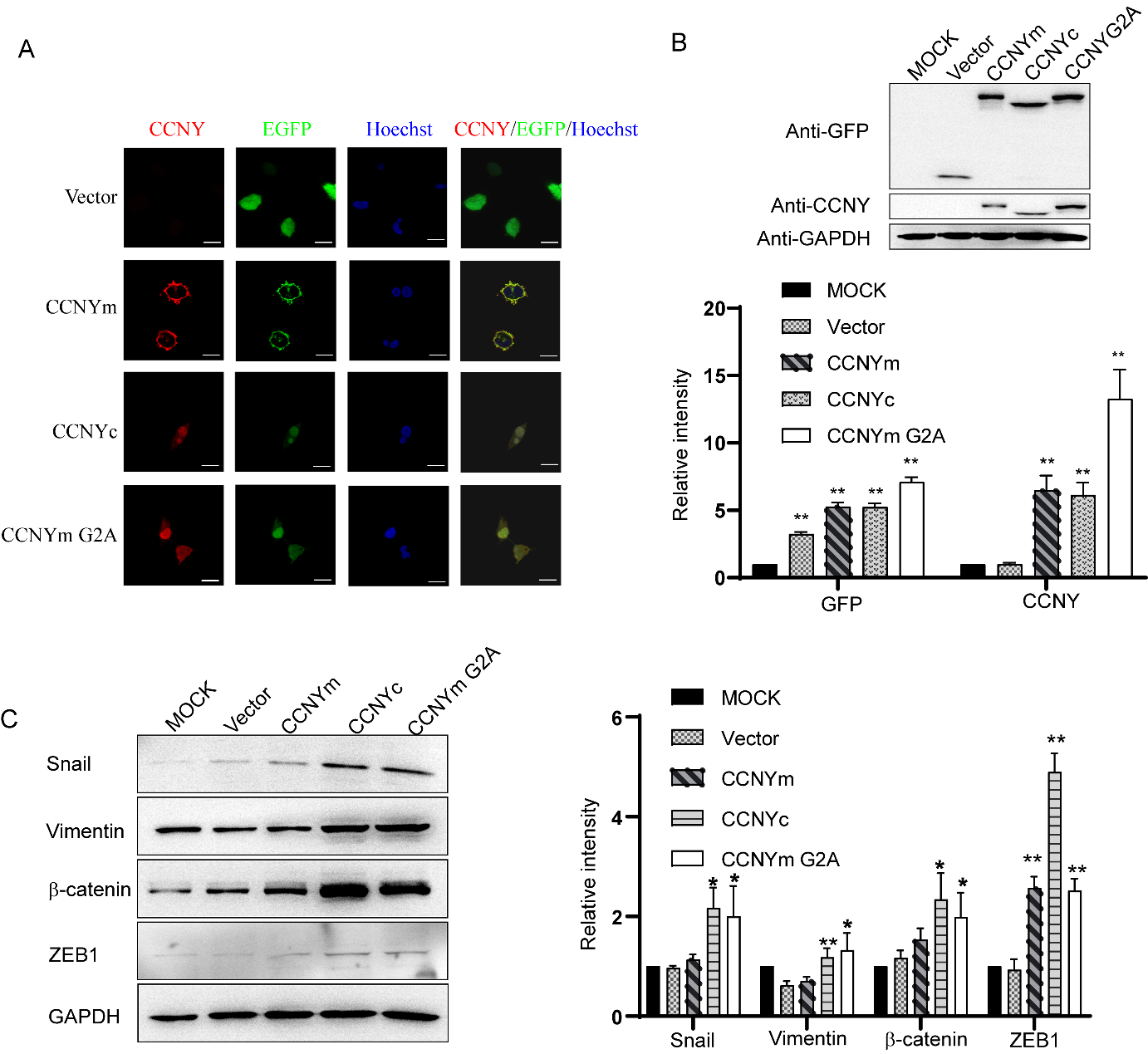


**Supplementary Figure 4** EMT was caused by the expression of CCNYm in HEK293 cells. A, HEK293 cells were transfected with pEGFP-N1-CCNYc, pEGFP-N1-CCNYm, pEGFP-N1, and pEGFP-N1-CCNYm G2A. Expression levels of GFP and CCNY were examined by immunofluorescence assay. Bars represent 20 μm. B, CCNY level of HEK293 cells were determined by western blot. The lower panel was the quantification of CCNY. ** p <0.01 vs. HEK293 cells. C, Expression of ZEB1, snail, β-catenin and vimentin in CCNY upregulated HEK293 cells was determined by immunoblotting (left panel). The quantification was done by grey analysis shown in the right panel. * p <0.05 vs. HEK293 cells transfected with the pEGFP-N1 vector. ** p <0.01 vs. HEK293 cells transfected with the pEGFP-N1 vector.


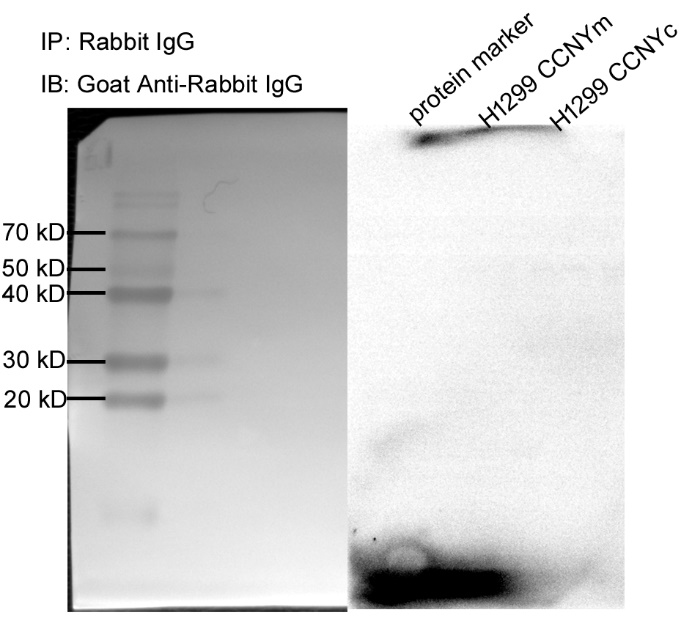


**Supplementary Figure 5** Negative control immunoprecipitation assay. Rabbit IgG was used as the capture antibody in the Co-IP assay. Rabbit polyclonal to PFTK1 antibody was used as detection antibody. The result was shown in the figure. The left panel was the photo of the membrane with white light, the right panel was the photo of the membrane with ECL No band appeared in the membrane.


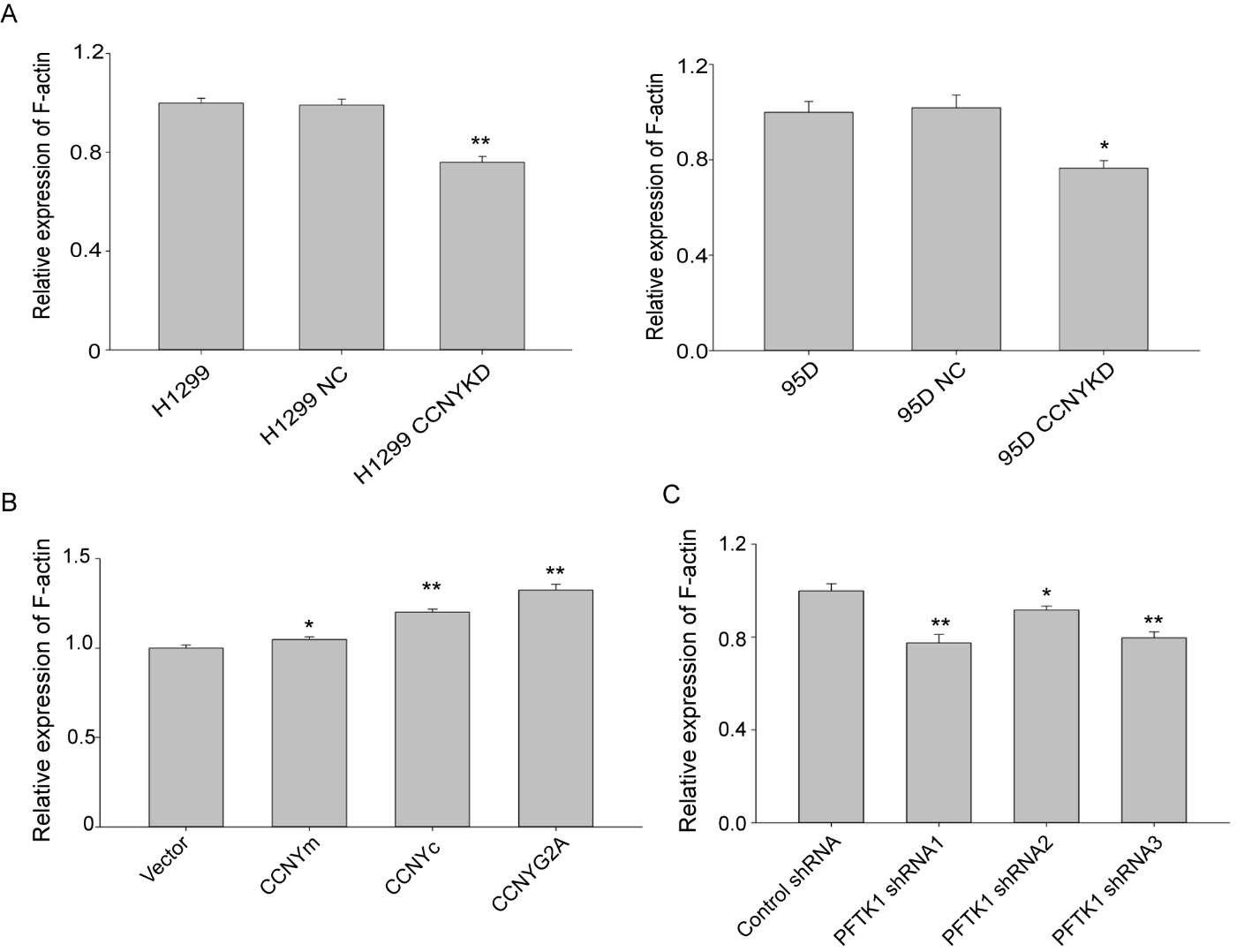


**Supplementary Figure 6** F-actin level was regulated by CCNY in H1299 cells. A-C, the F-actin was analyzed by high-content cell analysis. The data are expressed as mean ± SEM.*p <0.05, ** p <0.01 vs. NC cells.


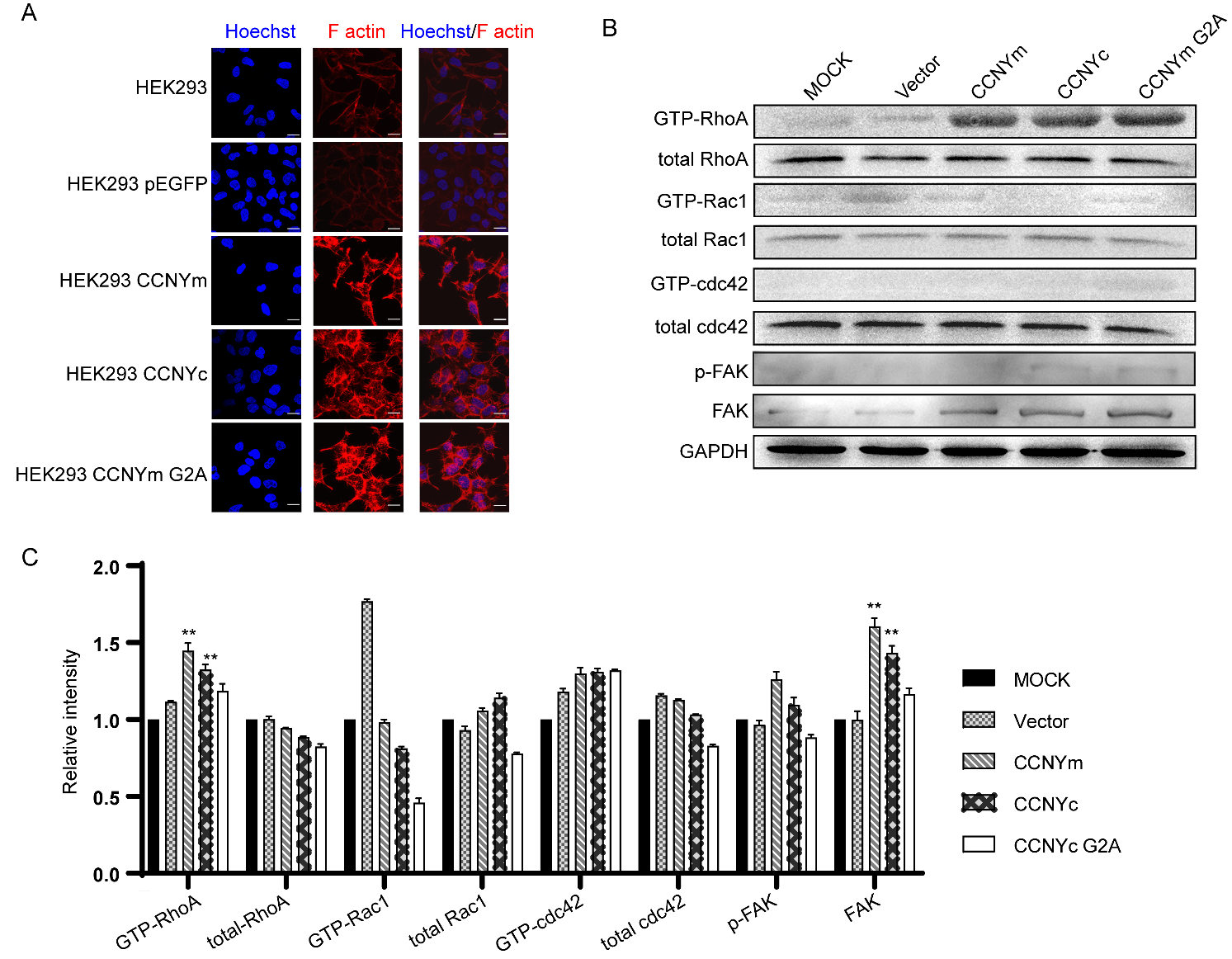


**Supplementary Figure7** F-actin level and RhoA activity were regulated by CCNY. A, F-actin was labeled by IF. Bars represent 20 μm. B, GTPase pull-down assay was used to detect the GTP-bound Rac1, RhoA, and Cdc42. The phosphorylated FAK on Y397, as well as the FAK level, were detected by immunoblotting. The quantification was done and showed as the left panel. ** p <0.01 vs. HEK293 cells transfected with the pEGFP-N1 vector.
